# Differences in maternal and paternal pressure to eat and perception of household responsibilities

**DOI:** 10.1371/journal.pone.0302331

**Published:** 2024-04-25

**Authors:** Paula Patel, Anusha Samant, Kychelle Del Rosario, Mara Z. Vitolins, Joseph A. Skelton, Edward H. Ip, Caroline B. Lucas, Callie L. Brown

**Affiliations:** 1 Wake Forest University School of Medicine, Winston-Salem, NC, United States of America; 2 Wake Forest University, Winston-Salem, NC, United States of America; 3 Department of Epidemiology and Prevention, Wake Forest University School of Medicine, Winston-Salem, NC, United States of America; 4 Department of Pediatrics, Wake Forest University School of Medicine, Winston-Salem, NC, United States of America; 5 Department of Biostatistics, Wake Forest University School of Medicine, Winston-Salem, NC, United States of America; Faculty of Medicine, University of Belgrade, SERBIA

## Abstract

Controlling feeding practices, such as pressure to eat, are associated with a child’s disinhibited eating and extremes in bodyweight. We aimed to explore which factors are associated with parent dyads’ pressuring feeding practices, including how mothers and fathers perceive the sharing of household tasks such as mealtime and child feeding responsibilities. In this cross-sectional study, parent dyads (mother and father) of healthy preschool-aged children completed an identical questionnaire consisting of measures of picky eating (food fussiness subscale of Child Eating Behavior Questionnaire), parental concern for undereating, and pressure to eat (Child Feeding Questionnaire). We used separate multivariable linear regression models for mothers and fathers to assess correlates associated with pressure to eat subscale score, including slowness of eating and enjoyment of food, child BMI z-score and race/ethnicity, and household income. Separate unadjusted linear regression models for mothers and fathers were used to report the association of pressure to eat with household responsibilities. Parents (N = 88) had similar mean picky eating, concern for undereating, and pressure to eat scores; more fathers had high pressure to eat scores (36% vs 27%). Higher pressure to eat was significantly associated with lower income, non-Hispanic Black or Black race/ethnicity, slow eating, and lower enjoyment of food. Pressure was not associated with household responsibilities. While there were similar maternal and paternal perceptions of child eating behaviors, more fathers reported pressuring their child to eat. Identifying differences in parental feeding practices may assist in intervention development to improve feeding practices.

## Introduction

As the prevalence of childhood obesity and chronic disease is steadily rising nationwide, contributing factors are being explored as possible intervention targets. Parents have a strong influence on their child’s environment and impact on their child’s eating habits, especially at a younger age [1]. For this reason, it is important to assess parental feeding practices as previous research has shown that certain feeding practices, such as restrictive behaviors, lead to increased weight gain in children by teaching children to respond to external cues as opposed to internal cues [2,3].

While parental restriction of a child’s food/eating has been shown to increase child food consumption, pressuring children to eat has been less studied. One study simulated an environment in which a child was pressured to eat and concluded that when children were not pressured to eat, they would eat more [4]. Parental pressure to eat has been associated with factors such as socioeconomic status, race, and parental education level [5], and has also been associated with maternal concern about picky eating and undereating [6]. These same concerns need to be examined in fathers, as research has shown that fathers have a significant gap in knowledge of child development, especially in nutrition [7]. This absence in knowledge could negatively affect feeding practices and child weight, especially as fathers’ roles in the home and childcare are evolving. As fathers take on a more involved role in the home and childcare [8], they are more likely to participate in their child’s mealtime; therefore, it is crucial to assess their feeding practices. Previous research has shown that mothers’ influences exert strong control over a child’s weight [9], but the role of fathers’ influences is less clear. Additionally, as fathers take on greater household responsibilities, it is unknown how mothers and fathers perceive the sharing of household tasks with changing gender roles. Previous research has shown that increased home demands can lead to conflict in the household [10]. It is unclear how parents of young children perceive the division of household responsibilities related to feeding, and whether these perceptions are associated with feeding practices. Therefore, the goal of this study is to explore mother’s and father’s report of pressuring their child to eat and to evaluate which child and family factors are associated with parents’ pressuring feeding practices, including mothers’ and fathers’ perceptions of the sharing of household tasks, such as mealtime and child feeding responsibilities.

## Subjects, materials, and methods

### Study overview

We performed a cross-sectional sub-study from the baseline data of a larger prospective cohort study assessing parental feeding practices in parents of healthy children [11]. Participants were recruited from four pediatric clinics affiliated with Atrium Health Wake Forest Baptist, including a resident continuity clinic, urban clinic, rural clinic, and suburban clinic. Children between the ages of 3–5 years and their parent were recruited between February 20, 2020 and January 21, 2021. The study was approved by the Wake Forest University School of Medicine Institutional Review Board.

Children were excluded from the larger study if the child was born prematurely (<37 weeks gestation); had a birth weight <2500 g; had a chronic medical problem affecting weight gain patterns or prompting special diets (e.g., congenital heart disease, renal disease, food allergies); or had a documented feeding/eating disorder, developmental delay, or intellectual disability (including autism). Only one child per family was eligible for enrollment; if two siblings were eligible, the parent was approached about the younger sibling’s participation.

The study was introduced to the parent who accompanied the child to the appointment and written informed consent was obtained by trained study personnel in the clinic examination room. The parent was asked to complete a survey on paper or on a tablet using REDCap software. Study personnel offered to read the survey to address issues of low literacy; however, no parents utilized that option. As part of the survey, parents reported all family members living in the household. The parent received a $10 gift card for completing the study.

For this sub-study, we contacted the other parent in the household (as applicable) by phone between and May 6, 2020 and March 10, 2021 and asked them to participate in the study as well. All eligible parents were contacted at least once and a voicemail was left, if available, with return contact information. Verbal consent was obtained, and an identical survey was administered to that adult over the phone. Responses were recorded into REDCap by a study team member. These participants were not paid for their participation in the study.

### Measures

The survey measured various aspects of child eating behaviors including picky eating, parental concern for undereating, pressure to eat, and relevant covariates. Picky eating was assessed using the 6-item Food Fussiness subscale of the Child Eating Behavior Questionnaire (CEBQ) [12]. Parents responded on a 5-point scale, and responses were averaged (range 1–5 with higher scores indicating higher picky eating). The Food Fussiness subscale is the most commonly used measure of picky eating [13] and has validity and good internal consistency both in more affluent predominantly white populations [12] and in low-income populations of young children in the United States (Cronbach’s α = 0.88) [14]. Additionally, a subscale from the Child Feeding Questionnaire (CFQ) was used to assess parental pressure to eat. Parents responded on a 5-point scale, and the scores were averaged. The pressure to eat subscale (4 items) of the CFQ has high internal consistency (0.7) [15] and is validated in non-Hispanic white [15] and non-Hispanic Black and Hispanic populations [16]. An average pressure to eat subscale score >3 is considered high pressure to eat. Child eating behaviors were assessed using four subscales of the CEBQ: satiety responsiveness, food responsiveness, enjoyment of food, and slowness in eating, using a 5-point scale (1 = never; 5 = always). These subscales have demonstrated validity and good internal consistency both in more affluent predominantly white populations [12] and in young Black and Hispanic children in the United States(Cronbach’s α = 0.70 for satiety responsiveness; 0.75 for enjoyment of food; 0.76 for food responsiveness; and 0.77 for slowness in eating) [17,18]. Parental concern for undereating was assessed using the prompt “I am concerned that my child doesn’t eat enough,” with responses on a 5-point scale (1 = strongly disagree; 5 = strongly agree); responses are considered positive if parents agree or strongly agree [19].

Questions involving the sharing of household responsibilities were adapted from the “Who Does What” questionnaire to assess the involvement of each parent in the child’s feeding practices [20]. Using a 9-point scale, parents were asked how much of a task (grocery shopping, planning and preparing meals, etc.) they were likely to perform. A response of 1 indicates that “My partner or other household member does it all,” a response of 5 indicates “We both do this about equally,” and a response of 9 indicates “I do it all.” Questions specific to child feeding (i.e., deciding where and when the child eats, and feeding/offering food to the child at mealtime) were added to this survey. We summed the mother and father responses to obtain a total dyad score (possible range 2–18) in which a score of 10 would indicate perfect agreement between dyads and scores at the extremes indicate the dyad believing they do more (scores higher than 10) or less (scores less than 10) of the total responsibility. We dichotomized the dyad scores to indicate agreement (total score 9–11) or disagreement (score <9 or >11).

Demographic information was obtained from the parent who completed the initial survey in the clinic, including household income (categorized in this analysis as <$60,000 vs $60,000+); parent education; their child’s sex, race and ethnicity (categorized in this analysis as non-Hispanic Black, non-Hispanic white, or Hispanic; no “other” race category options were reported for this sample), and number of siblings. Household food insecurity was assessed using the 2-item screen by Hager, et al. [21]. An affirmative response to either question is a positive screen and has high sensitivity (97%) and specificity (83%) compared to the gold-standard 18-item United States Department of Agriculture Household Food Security Survey [22]. Neighborhood safety was assessed with the Built Environment Safety Scale, a 9-item measure assessing overall neighborhood safety, traffic, and crime that was designed for use in parents of preschool children [23].

The child’s height, weight, date of visit, and date of birth were obtained from the child’s electronic medical record. Using this information, body mass index (BMI) percentiles, and BMI z-score were derived using the Center for Disease Control and Prevention reference growth charts for age and sex [24]. Weight status was subsequently classified as underweight (BMI <5th percentile for age and sex), healthy weight (5th to <85th percentile), overweight (85th to <95th percentile), and obesity (≥95th percentile).

### Statistical analysis

Descriptive analyses were performed and normality was assessed using histograms. There were no extreme values. Normality was also assessed using the Shapiro-Wilk test and continuous variables were normally distributed. Participant characteristics of the cohort used in this analysis was compared to the original study’s larger cohort by using Fisher’s exact or a t-test for categorical or continuous variables, respectively. Bivariate analyses were performed using unadjusted linear regression analyses to separately examine associations of maternal and paternal pressure to eat (continuous variable) with child sex (male or female), age (continuous), and race/ethnicity (categorical) as reported by the original parent participating in the main study; the child’s BMI z-score; each parent’s report of the child’s picky eating (continuous), concern for undereating (categorical), satiety responsiveness (continuous), slowness of eating (continuous), food responsiveness (continuous), and enjoyment of food (continuous); and household income (categorical) and food insecurity (categorical). We used separate mutivariable linear regression models for mothers and fathers to assess correlates associated with pressure to eat subscale score, including slowness of eating and enjoyment of food, child BMI z-score and race/ethnicity, and household income. Mother and father dyads were then designated as having concordant pressuring feeding practices if both parents were high- or low-pressure feeders and were designated as being different if one was high and one was low. Descriptive statistics were used to describe parents’ concordant feeding practices. Descriptive statistics were also used to report the sharing of household responsibilities between mothers and fathers. Separate unadjusted linear regression models for mothers and fathers were used to report the association of pressure to eat with each of the household responsibilities. Data analysis was performed using Stata v14.0 (College Station, Texas) using an alpha<0.05 for statistical significance.

## Results

Of the 335 participants in the initial study, 245 had another adult living in the household. We completed telephone surveys for 46 other household adults, 7 of which were mothers, 37 fathers, 1 boyfriend/girlfriend of the biological parent, and 1 grandparent. For this analysis, we opted to only examine mothers’ and fathers’ data. Our final sample consisted of 44 households with a child, mother, and father triad who completed the survey, for a total of 132 participants and 88 parent survey respondents. Independent variables demonstrated no collinearity with all variance inflation factors <1.3 (mean 1.2).

Children were 39% male, racially diverse (59% non-Hispanic white, 27% non-Hispanic Black, 14% Hispanic), and 75% had a healthy weight. Households were diverse with respect to annual household income (21% <$20,000, 32% $20,000–60,000, and 48% at least $60,000). About half of households had 4 members, and 14% of households reported having food insecurity (Table 1). Compared to the original cohort (N = 330 child/parent dyads) the child/mother/father triads in this study had higher rates of high income (48% vs 25%) and non-Hispanic white race/ethnicity (59% vs 36%), but children and households were similar with respect to other characteristics.

**Table 1:** Characteristics of preschool-aged child participants and their households.

	N (%) or Mean (Standard Deviation)
Child Characteristics (N = 44)	
Male sex	17 (38.6%)
Race/Ethnicity	
Non-Hispanic white	26 (59.1%)
Non-Hispanic Black	12 (27.3%)
Hispanic	6 (13.6%)
BMI z-score	0.28 (1.22)
Child Weight Status	
Underweight	2 (4.6%)
Healthy Weight	32 (74.4%)
Overweight	4 (9.3%)
Obesity	5 (11.6%)
Household Characteristics (N = 44)	
Household Income $60,000+	21 (47.7%)
Household Food Insecurity	6 (13.6%)

Mothers and fathers both had a mean picky eating score for their child of 2.9 (p-value = 0.3). Mothers and fathers similarly expressed concern for their child’s undereating 11% of the time. The mean (standard deviation [SD]) pressure to eat score for mothers was not significantly different from fathers (2.6 [0.9] vs 2.8 [1.2], respectively, p = 0.2). However, less than a third (27.3%) of mothers self-reported that they place high pressure on their child for eating, compared to 36.4% of fathers, p = 0.02.

When examining factors associated with pressure to eat in bivariate analyses, neither maternal nor paternal pressure to eat was associated with child sex, household income, household food insecurity, or neighborhood safety (Table 2). Mothers had higher rates of high pressure to eat if the child was of Hispanic or non-Hispanic Black race/ethnicity or if the mother was concerned about the child not eating enough. Mothers who exerted high pressure to eat had children with higher mean picky eating, satiety responsiveness, and enjoyment of food scores. Fathers who exerted high pressure to eat had children with higher mean slowness in eating scores (Table 2).

**Table 2:** Factors associated with maternal and paternal high pressure to eat.

	Maternal N = 44 N (%) or Mean (Standard Deviation)			Paternal N = 44 N (%) or Mean (Standard Deviation)		
	High Pressure	Low Pressure	p-value	High Pressure	Low Pressure	p-value
Child Characteristics						
Male sex	5 (42%)	12 (38%)	1.0	5 (31%)	12 (43%)	0.5
Race/Ethnicity			0.002			0.3
Non-Hispanic white	3 (25%)	23 (72%)		7 (44%)	19 (68%)	
Non-Hispanic Black	4 (33%)	8 (25%)		6 (38%)	6 (21%)	
Hispanic	5 (42%)	1 (3%)		3 (19%)	3 (11%)	
BMI z-score	-0.04 (1.2)	0.4 (1.2)	0.3	0.4 (1.5)	0.2 (1.1)	0.6
Household Characteristics						
Household Income $60,000+	5 (42%)	16 (50%)	0.7	6 (38%)	15 (54%)	0.4
Household Food Insecurity	1 (8%)	5 (16%)	1.0	1 (6%)	5 (18%)	0.4
Neighborhood Safety Score	7.8 (2.6)	6.9 (2.6)	0.4	7.4 (2.3)	8.3 (2.6)	0.3
Child Feeding Characteristics						
Picky Eating	3.5 (0.9)	2.7 (0.8)	0.01	2.8 (0.7)	2.9 (0.7)	0.9
Concern for Undereating	4 (33%)	1 (3%)	0.02	3 (19%)	2 (7%)	0.3
Satiety Responsiveness	3.4 (0.7)	2.6 (0.6)	<0.001	3.1 (0.8)	2.7 (0.5)	0.05
Slowness in Eating	3.3 (1.1)	3.0 (0.7)	0.2	3.6 (1.0)	2.8 (0.8)	0.008
Food Responsiveness	2.5 (0.7)	2.5 (1.1)	0.8	2.6 (0.7)	2.7 (0.9)	0.9
Enjoyment of Food	3.5 (0.8)	4.1 (0.6)	0.009	3.9 (0.6)	4.1 (0.5)	0.3

When examining factors associated with pressure to eat in multivariable analyses, household income was not associated with either maternal or paternal pressure to eat (Table 3). For mothers, a higher pressure to eat score was associated with the child’s Hispanic or non-Hispanic Black race/ethnicity and the child’s enjoyment of food score. For fathers, pressure to eat was associated with the child’s slowness of eating (Table 3).

**Table 3:** Factors associated with maternal and paternal pressure to eat.

	Maternal Pressure Beta (95% CI)	Paternal Pressure Beta (95% CI)
Child Race/Ethnicity		
Non-Hispanic white	Ref	Ref
Non-Hispanic Black	0.75 (0.23, 1.27)**	0.03 (-0.84, 0.91)
Hispanic	1.45 (0.81, 2.09)***	-0.04 (-0.96, 1.03)
Child BMIz Score	-0.19 (-0.37, -0.002)*	0.07 (-0.21, 0.36)
Household Income $60,000+	-0.40 (-0.83, 0.03)	-0.54 (-1.23, 0.15)
Enjoyment of Food	-0.38 (-0.68, -0.08)**	-0.54, (-1.20, 0.12)
Slowness of Eating	0.17 (-0.10, 0.44)	0.58 (0.22, 0.93)**

* represents p<0.05

** p<0.01

*** p<0.001.

Mothers and fathers had concordant pressuring feeding practices 73% of the time. When parents had differing pressuring practices, fathers had high pressuring practices 18% of the time and mothers had high pressuring practices 9% of the time. When mothers had high pressuring feeding practices, the fathers also had high pressure 67% of the time. When fathers had high pressuring feeding practices, the mothers also had high pressure 50% of the time.

**Fig 1:**
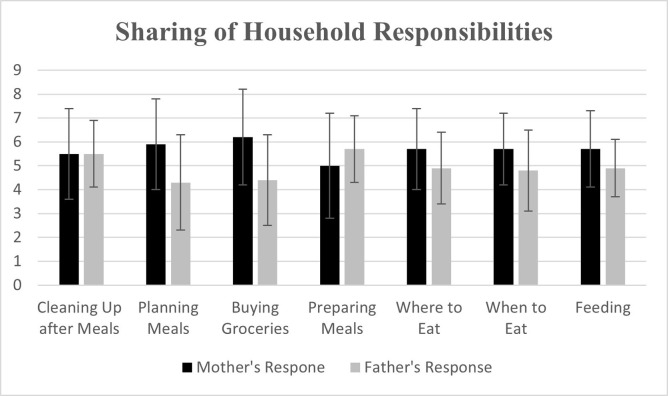
Parent perception of how much of the task the parent believed they performed, with 1 being “My partner or other household member does it all,” 5 being “we both do this about equally,” and 9 being “I do it all”.

Finally, the sharing of household responsibilities between mothers and fathers was reported by each parent (scale 1–9). As displayed in Fig 1, mothers reported performing more of the household tasks compared to fathers. However, in respect to all tasks, the mean combined score of parents added up to >10 (total score should be 10 or less), indicating disagreement and incongruence about how much responsibility each parent had for a single task and each parent thought they did more than their partner reported that they did. For instance, for the task of planning meals, mothers reported a mean score of 5.9 whereas fathers mean score was 4.3, leading to a combined completion of the task as a 10.2. Mothers reported a mean score of 5.7 for feeding/offering food to their child at mealtimes, while fathers reported a mean score of 4.9 for a combined score of 10.6. Other combined scores for cleaning up after meals, buying groceries, or preparing meals were 11.0, 10.6, and 10.7, respectively. Parents frequently disagreed about the sharing of household responsibilities: cleaning up after meals (36% of parents disagreed), planning meals (43%), buying groceries (32%), preparing meals (45%), deciding where to eat (30%), deciding when to eat (39%), and feeding/offering food to their child at mealtimes (32%). Maternal and paternal pressure to eat were not associated with household responsibilities for any of the household tasks. For example, mothers’ perception of their responsibility for feeding the child was not associated with maternal pressure to eat (β 0.10 [95% CI -0.07, 0.27]) or paternal pressure to eat (β 0.01 [95% CI -0.21, 0.23]) and fathers perception of their responsibility for feeding the child was similarly not associated with maternal pressure to eat (β -0.04 [-0.28, 0.20]) or paternal pressure to eat (β 0.22 [-0.07, 0.52)]).

## Discussion

This study assessed concerns about child eating and feeding practices, including pressure to eat, among triads of mothers and fathers of preschool-aged children. Our findings show that mothers and fathers had similar perceptions of their child’s picky eating and concern for their child’s undereating. While mean pressure to eat scores were similar between mothers and fathers, more fathers reported using high pressure feeding practices compared to mothers. In multivariable analysis, maternal pressure to eat was significantly associated with non-Hispanic Black or Hispanic race/ethnicity, child BMIz, and lower enjoyment of food while paternal pressure was associated with the child’s slow eating. Both mothers and fathers reported being significantly engaged in all aspects related to their child’s feeding, however, parents’ report of household responsibilities demonstrated disagreement and incongruence regarding the amount of responsibility shared between both parents for certain household tasks.

Consistent with previous research, mothers demonstrated a significant association between higher enjoyment of food with lower pressure to eat. A research study looking at methods to decrease picky eating in children showed that pressure had an indirect effect on picky eating through eating enjoyment [25]. Other studies also show that a higher enjoyment of food score is correlated with a higher child’s BMI z-score; therefore, similar reasons likely exist for why mothers may not pressure children who enjoy eating food [26].

The only factor that had a significant association with fathers’ pressure to eat was concern about the child’s slowness of eating, which was associated with increased pressure. Slowness of eating likely increases the time that fathers spend at mealtime with the child, and it may lead to tactics to hurry along the process. Fathers have been noted to employ increased pressure to get children to eat faster [27]. This may contribute to increased childhood obesity as it undermines a child’s response to his or her internal cues of hunger and satiety [28]. Research shows slower eating is an indication of heightened responsiveness to satiety [26]. Therefore, pressuring a child to eat quickly and to eat when he or she is not hungry can lead to overconsumption of food. Education of parents, including fathers, about child feeding behaviors could potentially prevent this. Slowness of eating was not seen as a factor that was associated with maternal pressure to eat, which may be attributed to mothers being more patient as potentially they spend more time with children and are able to recognize their behaviors [29]. Moreover, mothers are often the caregivers most responsible for taking their young child to medical appointments where they receive counseling on healthy feeding practices. This education promotes more healthful ways of feeding, as research has shown there to be a positive relationship between anticipatory guidance on feeding practices and healthy eating and growth patterns in children [30,31]. Encouraging families to develop consistent feeding practices is important, as congruent parenting by mothers and fathers is associated with healthier child eating practices [32].

Finally, our study looked at the difference in perception of who completes what household tasks between mothers and fathers. Both mothers and fathers reported being significantly engaged in all aspects related to their child’s feeding, however, parents’ report of household responsibilities demonstrated disagreement and incongruence regarding the amount of responsibility shared between both parents for certain household tasks. There was disagreement about the extent of shared responsibility for every task. If both parents feel that they are doing more, it may create a stressful environment and conflict in the home. Previous studies have shown that when fathers engage in more child-care tasks, the family as a whole is closer, promoting a better home environment [10]. Clinicians can assist by discussing the importance of sharing household responsibilities equally. More research needs to be conducted in this area to determine whether incongruence between parents’ perceptions on household task responsibilities has an impact on children’s eating habits and their health.

While fathers historically have had a minimal role in child and household caretaking responsibilities, many are more recently taking on an increased role; 17% of stay-at-home parents are now fathers [33]. Despite fathers taking a larger role in childcare, healthcare facilities have not yet shifted to support this change. Pediatric providers have not changed their practices to address fathers’ unique concerns regarding their children [34]. Therefore, counseling focused on education of all family caregivers should be explored to optimize the best care for children. Practices such as telemedicine and providing father-specific pamphlets on feeding practices can be employed to promote education. Developing alternative strategies for delivering this information is especially important as mothers continue to be the caregiver most commonly attending visits with pediatric providers, as was seen in our study.

Study limitations should be considered when interpreting these results. This study was performed at pediatric primary care clinics associated with a single institution and within a single geographic region in North Carolina. Additionally, this study only included English-speaking participants, limiting the generalizability of these findings to other populations. Our study only looked at mothers and fathers living in the same household, which may have contributed to including more socioeconomically-advantaged fathers who are in a positive relationship with the child’s mother [35]. Our study was also limited by the small percentage of fathers who we were able to reach by phone and complete the survey, which could lead to inadequate power (especially for subgroup comparisons) and limit the generalizability of our findings. We also did not have information on parental employment, which could affect the division of household responsibilities. Furthermore, we were not able to examine other family structures, such as a mother and father co-parenting but not living in the same house or same-sex parents. Finally, participants were asked to self-report information, which can lead to social desirability bias. Future work should consider using objective and unbiased assessments of parent feeding practices and household tasks.

## Conclusions

While mothers and fathers had similar perceptions of their child’s eating behaviors, fathers had increased rates of high pressure to eat feeding practices. It may be necessary to place more effort on assessing both parents’ feeding practices as parents often utilize different practices even for the same child. Identifying differences in parental feeding practices may assist in the development of interventions targeting all members of the family to improve their feeding practices.

## Supporting information

S1 Checklist (DOCX)
